# Prevalence of echinococcosis and *Taenia hydatigena* cysticercosis in slaughtered small ruminants at the livestock-wildlife interface areas of Ngorongoro, Tanzania

**DOI:** 10.14202/vetworld.2017.411-417

**Published:** 2017-04-19

**Authors:** M. B. Miran, A. A. Kasuku, E. S. Swai

**Affiliations:** 1 Department of Livestock, Ngorongoro District Council, P. O. Box 1, Loliondo, Tanzania; 2 Department of Microbiology and Parasitology, Faculty of Veterinary Medicine, Sokoine University of Agriculture, P.O. Box 3015, Morogoro, Tanzania; 3 Ministry of Agriculture, Livestock and Fisheries, P. O. Box 9152, Dar-es-Salaam, Tanzania

**Keywords:** cysticercosis, *echinococcosis*, small ruminants, Tanzania, wildlife interface

## Abstract

**Aim:**

Echinococcosis or hydatidosis (due to the larval stage of *Echinococcus* spp.) and cysticercosis (due to the larval stage of *Taenia hydatigena*) pose a significant economic losses due to slaughter condemnation and risk to public health in developing countries such as Tanzania where sanitation is poor and people live in close proximity with each other and with animals. This study was conducted to determine the prevalence of and to identify the predisposing factors for echinococcosis and *cysticercosis* in sheep and goats at three slaughter slabs located in the livestock-wildlife interface areas of Ngorongoro, Tanzania.

**Materials and Methods:**

A cross-sectional based survey was conducted, from January 2013 to April 2013, whereby a total of 180 animals comprising 90 goats and 90 sheep of both sexes were examined at postmortem for the evidence of larval stages of *Echinococcus* spp. (hydatid cyst) and *T. hydatigena* (*Cysticercus tenuicollis*) through visual inspection, incision and palpation of organs and viscera.

**Results:**

The prevalence of echinococcosis was 22.2% and 16.6%, in goats and sheep, respectively, while the overall infection rates for cysticercosis were 61.1% in goats and 42.2% in sheep. The result of this study revealed that goats and sheep in Malambo slaughter slab had significantly higher prevalence of *T. hydatigena* (*C. tenuicollis*) and hydatid cysts (p<0.05) compared to other slab points. *T. hydatigena* (*C. tenuicollis*) *cysts* were more frequently detected in the omentum than other visceral organs among the animals examined.

**Conclusion:**

In conclusion, the observed high prevalence of the two metacestodes larval stages leads to high condemnation rates of edible offals and raises significant public health concerns. This underscores for the need to undertake more extensive epidemiological investigations to better determine the causal factors, economic impact, and public health importance of the disease in this livestock-wildlife interface setting.

## Introduction

Goats and sheep represent the second and third largest proportion of the livestock population in Tanzania, respectively. Tanzania’s small ruminant wealth in 2008 included 15.1 million goats and 5.7 million sheep [1]. More than 99% of this livestock are kept in low-input low-output systems, owned and managed by 1,732,863 low income mixed and pastoral households who operate under traditional husbandry system, often with little or no access to informed and relevant animal production advice or reliable veterinary services.

Cestodes of the family *Taeniidae* infect carnivores as the definitive host and are transmitted to a wide range of intermediate host species where they cause hydatidosis and cysticercossis. *Cysticercus tenuicollis* and hydatid cyst are larval stages of the canine tapeworm *Taenia hydatigena* and *Echinococcus granulosus*, respectively. The two cestodes are found in a large number of hosts throughout the world [2-5]. The cystic echinococcosis (also called hydatidosis) and *T. hydatigena* cysticercosis (due to *C. tenuicollis)* are widespread parasitic diseases infecting a large number of wild and domestic animals and humans and are considered as one of the major causes of economic losses and productivity of livestock in both the developing and industrialized world [6-8]. The intermediate host contracts infection by ingesting proglottids or eggs passed in dog feces that contaminate the pasture or feeding areas [3]. Cysts can be spread by other canids such as wolves, jackals, and foxes which are definitive hosts [9]. The loss due to the condemnation of organs by hydatid and *C. tenuicollis* cysts in small ruminants is significant in countries, such as Tanzania, where there are low standards of sanitation, unregulated home slaughter and close contact between people and animals especially in pastoral communities [10].

Although numerous surveys on the prevalence have been reported in different parts of the world [10,11], there are a very limited number of studies on *C. tenuicollis* and hydatid cyst in Tanzania [12,13]; no comprehensive study has been conducted of these parasites in sheep and goats in the livestock-wildlife interface. Analysis of the factors influencing the occurrence of these parasites might provide further insight into the epidemiology of *C. tenuicollis* and hydatid cyst infections. Because of the scarcity of such data in the literature, we aimed by the present work, to determine the prevalence of hydatidosis and cysticercosis in sheep and goats as well as to explore the influence of sex, age and slaughter slab location as determinants of infection.

## Materials and Methods

### Ethical approval

Permission to carry out this study was granted by the District Executive Director of Ngorongoro. The protocols for this study were approved by the Sokoine University of Agriculture, Morogoro, Ethics Committee. Before starting data collection, and after explaining the purpose and importance of the study, the consent was obtained from each of the trade stock owners to conduct the necessary carcass examinations. Operating procedures regarding the safety of researchers, community and environment were strictly adhered to at all stages of sample collection, handling and processing.

### Study area

**Figure-1:**
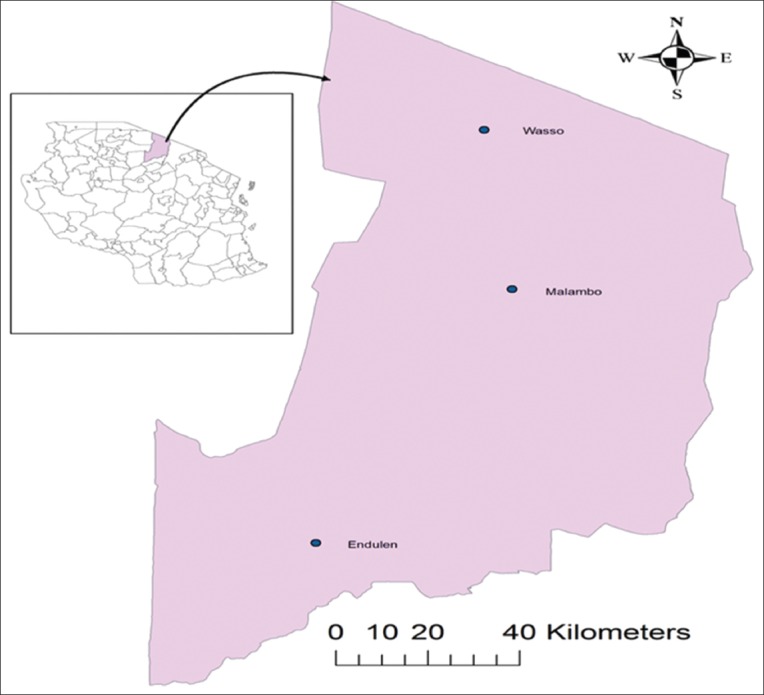
A map of Ngorongoro district showing the study villages. The insert is map of Tanzania.

This study was conducted in Ngorongoro, the largest of the seven districts in Arusha region, northern Tanzania. The district lies between longitude 35’30° and 36’23° E and latitude 02’45° and 4’01° S covering an area of 14,036 km^2^ (Figure-1). Geographically, the district is bordered by Kenya to the north, Serengeti National Park to the west, Longido and Monduli Districts to the east and Karatu District to the south. The district has heterogeneous physical and climatic features varying from cool (Loliondo and Ngorongoro Conservation Area Authority) highlands in the North and South, respectively, to semi-arid plains in the central West and South. Administratively, it is divided into 3 divisions, 21 wards and 43 registered villages and subvillages. Other climate and weather related information are described in detail by Miran *et al*. [14].

### Participating slaughter slab and population of interest

Three slaughter slabs located in Waso, Malambo and Endulen villages were selected in collaboration with the government livestock extension and administration officers. Selection criterion of the villages was based on the high numbers of slaughter of sheep and goats at these sites. Only animals originating from within the village and the surrounding area where a particular slaughter slab is located were examined during the study. To avoid or minimize the inclusion of slaughtered animals originating from outside the study area, animals slaughtered during livestock market days were excluded in the study. The study was conducted from January 2013 to April 2013. In each month, about 50 animals (25 sheep and 25 goats) of both sexes and of different age groups (classified as ≤1 to 1, ≥1 to 2, ≥2 years) slaughtered at three local slaughter slabs (Waso, Malambo, and Endulen) were randomly selected and individually identified.

### Parasitological examination

The presences of tape-worm metacestodes (specifically *C. tenuicollis* and hydatid cyst) in the carcass and/or internal organs were examined, and the results/findings were recorded. Particular attention was paid to the omentum, mesentery, peritoneal cavity, spleen, lung, and liver. The numbers and localizations of cysts were recorded.

### Data analysis

Data were coded and entered into a Microsoft Office Excel (spreadsheet) and analyzed using Epi Info 7 software. The prevalence was computed as the proportion of sampled animals which harbor cysts. The Chi-square test was used to assess the statistical difference between proportions. Fisher’s exact test was used when the number within categories were too small for the Chi-square. In all analyses, a critical probability of p<0.05 was used for statistical significance.

## Results

### Prevalence of metacestodes of *Tenia* and *Echinococcus* tapeworms

The prevalence’s of *E. granulosus* and *T. hydatigena* metacestodes in three slaughter slabs by species are as indicated in Table-1.

**Table-1:** The prevalence (with exact±95% CIs) of *C. tenuicollis* cysts and hydatid cysts by species (January 2013-March 2013).

Species	*C. tenuicollis* cysts		Hydatid cyst	
	
	Number positive	Prevalence % (±95% CI)	Number positive	Prevalence % (±95% CI)
Ovine	38	42.2 (32.3-52.6)	15	16.6 (9.6-26.0)
Caprine	55	61.1 (50.3-71.2)	20	22.2 (14.1-32.2)
Overall	93	51.7 (44.1-59.1)	35	19.4 (13.9-25.9)

CI=Lower and upper limits for 95% confidence interval of the prevalence, *C. tenuicollis=Cysticercus* *tenuicollis*

During postmortem examination, the larval form of *T. hydatigena* tape-worm (*C. tenuicollis*) was found attached to the mesenteries. The overall prevalence was 51.7% (95% confidence interval [CI]: 44.11%, 59.16%) and only 27.3% appeared as a single infection. The species prevalences were 61.1% and 42.2% in goats and sheep, respectively, as shown in Table-1. The prevalences in sheep and goats are statistically significant different (χ^2^=6.35, p=0.011).

Of 180 examined carcasses, 19.4% (95% CI: 13.93%, 25.99%) were infected by the larval form of *E*. *granulosus* (hydatid cyst) (Table-2), 16.6% were found in sheep, and 22.2% in goats. 40% and 22.9%, respectively, of the cysts were located only in the lungs and liver (Table-3). Concurrent infections were 25.7% in the liver and lungs; 2.9% in the lungs together with spleen and 8.6% in all three organs (liver, lungs, and spleen). No hydatid cysts were found in either the spleen or in the liver and spleen concurrently in sheep. The differences in the prevalence in the species and sex were both not statistically significant being χ^2^=0.82 (p=0.36) and χ^2^=0.323 (p=0.569), respectively.

**Table-2:** Prevalences of *T. hydatigena* (*C. tenuicollis*) metacestodes in sheep and goats in three slaughter slabs (n=90).

Variable	Categories	No examined	Proportion examined (%)	No positive	Prevalence (%)	χ^2^	p value
Sheep							
Sex	Male	60	66.6	24	40	0.36	0.546
	Female	30	33.4	14	46.7		
Slaughter slab	Waso	30	33.3	11	36.7	6.10	0.047
	Malambo	30	33.3	18	60		
	Endulen	30	33.3	9	30		
Age	<1 years	14	15.5	5	35.7	0.306	0.858
	>1-2 years	19	21.1	8	42.1		
	>2 years	57	63.3	25	43.8		
Goats							
Sex	Male	41	45.5	8	19.5	0.32	0.572
	Female	49	55.5	12	24.5		
Slaughter slab	Waso	30	33.3	5	16.7	8.61	0.013
	Malambo	30	33.3	12	40		
	Endulen	30	33.3	3	10		
Age	<1 years	22	24.4	11	50	2.78	0.250
	>1-2 years	20	22.2	15	75		
	>2 years	48	53.3	29	60.4		

T. hydatigena=Taenia hydatigena, C. tenuicollis=Cysticercus tenuicollis

**Table-3:** Prevalence and distribution pattern of hydatid cyst lesion in different organs in slab-slaughtered sheep (n=90) and goats (n=90) in Ngorongoro district.

Species	Organ location in percentage				

	Liver	Lungs	Liv&lun	Lun&spl	All
Ovine	20.0	46.7	26.7	0.0	6.7
Caprine	25.0	35.0	25.0	5.0	10.0
Total	22.9	40.0	25.7	2.9	8.6

Liv&lun - Liver and lungs, Lun&spl - Lungs and spleen, All - Liver, lungs and spleen, Total - Sheep and goats

### T. hydatigena infection

The prevalence of *T*. *hydatigena* metacestodes in sheep and goats by sex, age and slaughter slab are shown in Table-2. Slaughtered sheep and goats in Malambo were consistently associated with high prevalence as compared to Waso and Endulen (p<0.05). The variables of sex and age were not significantly associated with the prevalence of *C. tenuicollis* (p>0.05).

### Echinococcosis

The prevalence of hydatid cysts in sheep and goats by sex, age and slaughter slab are detailed in Table-4. Significantly, higher prevalences were detected in Malambo slaughter slab (p<0.05) for both species. In both animal species, age and sex were not significantly associated with prevalence (p>0.05).

**Table-4:** Prevalences of *Echinococcus* (hydatid cyst) metacestodes in sheep and goats in three slaughter slabs (n=90).

Variable	Categories	No examined	Proportion examined (%)	No positive	Prevalence (%)	χ^2^	p value
Sheep							
Sex	Male	60	66.6	10	16.7	0.02	1.00
	Female	30	33.4	5	16.7		
Slaughter slab	Waso	30	33.3	5	16.7	7.68	0.021
	Malambo	30	33.3	9	30		
	Endulen	30	33.3	1	3.3		
Age	<1 years	14	15.5	0	0	3.35	0.188
	>1-2 years	19	21.1	4	21		
	>2 years	57	63.3	11	19.3		
Goats							
Sex	Male	41	45.5	12	29.2	0.5	0.473
	Female	49	54.4	8	16.3		
Slaughter slab	Waso	30	33.3	5	16.7	8.61	0.013
	Malambo	30	33.3	12	40		
	Endulen	30	33.3	3	10		
Age	<1 year	22	24.4	1	4.5	3.82	0.148
	>1-2 years	20	22.2	5	25		

### Mixed infections/coexistence rates of metacestodes

A total of 128 (71.1%) carcasses were infected by different metacestodes (53 sheep and 75 goats). Of these, 11.5% (n=15) were mixed infections (Table-5).

**Table-5:** Mixed infection rates of *C. tenuicollis* and hydatid cysts lesions in animals slaughtered at Ngorongoro district (January 2013-March 2013).

Species	*C. tenuicollis* (%)	Hydatid cysts (%)	*C. tenuicollis* and hydatid cysts (%)
Ovine	42.2	16.6	9.4
Caprine	61.1	22.1	13.3
Total	51.7	19.45	11.5

C. tenuicollis=Cysticercus tenuicollis

## Discussion

Of 180 animals (90 sheep and 90 goats) examined in three slaughter slabs, 19.4% were found infected with hydatid cysts. This high prevalence was possibly because of the large dog population and the presence of other wild canids and the practice of home slaughter. Most of the slaughter slabs are not fenced and a dog helminth control strategy is not in place. Goats had the highest infection rate of 22.2% compared to 16.6% for sheep. However, the differences in species and by sex were not statistically significant, i.e. χ^2^=0.82 (p=0.36) and χ^2^=0.323 (p=0.569), respectively. The high prevalence found in goats recorded in this study is in agreement to the findings by Dalimi *et al*. [15] who reported a mean prevalence of 8.1% hydatidosis in sheep, 38.3% in goats in Iran and contrary to Oryan *et al*. [8] observation of 45.5%, and 10.0% in sheep and goats, respectively in Fars, southern Iran. Similarly, the finding of this study is contrary to Nyero *et al*. [16] observation of 42.5%, and 33.3% in sheep and goats, respectively, in Soroti, Uganda. The prevalence of hydatid cyst was higher in older sheep and goats (19.3% and 29.1% in >2 and >1-2 years, respectively) compared to the younger ones and higher in male goats (29.2%). This is in agreement with the study by Lahmar *et al* [17] in Tunisia and Helina [11] in Ethiopia who found higher prevalence in adult and males compared to young and female goats. Older animals have a more prolonged period of exposure and therefore much greater risk of infection and the chances of detecting cysts at meat inspection are higher in aged animals due to bigger size of the cysts [18]. Similarly, the previous studies strongly suggested that the prevalence is heavily influenced by age and origin of animals brought for slaughter [17,19].

In this study, the observed overall prevalence of hydatid cysts in goats and sheep is lower than that observed in temperate countries, i.e., Italy where a prevalence of 47-81.9% in sheep and 71.9% in goats were reported [20]. Higher prevalences were also observed in Eastern Ethiopia and North West Iran [21,22]. Although specific reason for such variations was not clear, illegal slaughter and uncontrolled dog population and movement was postulated to play a role.

About 40% and 22.9% of the hydatid cysts were located only in one organ namely the lungs and liver, respectively. As in other previous studies by Getaw *et al*. [6] in Central Oromia, Ethiopia and Ibrahim [23] in Al Baha region, Saudi Arabia, the lungs and liver were the organs most commonly affected by hydatid cysts in sheep and goats. The respective concurrent organ infections were 25.7% in the liver and lungs; 2.9% in the lungs together with spleen and 8.6% in three organs (liver, lungs, and spleen). Fromsa and Jobre [24] in Ethiopia abattoirs obtained the prevalences of 11.78% in sheep and 4.9% in goats. The prevalence of hydatid cysts from this study was lower than the findings obtained of 63.8% in sheep and 34.7% in goats by Ernest *et al*. [12] from Ngorongoro district. The differences may be attributed to the accuracy in the retrospective records and/or due to variations in the number of animals examined. However, in this study, the prevalence was higher than that reported by Nonga and Karimuribo [13] which was 6.02% in sheep and goats from Arusha. Although the prevalence was lower compared to the findings by Ernest *et al*. [25], still the prevalence suggests the possibility that hydatidosis is of public health importance in this study area and that some of the taeniid eggs recovered from the feces of dogs in the previous study may be eggs of *Echinococcus* species [26].

*T. hydatigena* metacestodes had the highest prevalence compared to those of *Echinococcus* spp. Out of the examined 90 sheep and 90 goats, the overall prevalence of *C. tenuicollis* was 51.7% and only 27.3% appeared as a single infection. Most of the cysticerci were found attached to the omentum or mesenteries.

The *C. tenuicollis* prevalence differences between sheep and goats were statistically significant (χ^2^=6.35, p=0.011) with the prevalence having been observed to be higher in goats (61.1%) compared to sheep (42.2%). The reason for higher prevalence of *C. tenuicollis* in goats than in sheep is not known given the differences in feeding behavior of these two species, i.e., browsers and grazers, respectively. However, similar observations were also documented in other studies by Radfar *et al*. [27] who reported a prevalence of 12.87% in sheep and 18.04% in goats in Iran; Nimbakar *et al*. [28] (goats 34.2% and sheep 21.4%) in Maharashtra, India; Bayu *et al*. [19] (goats 15.8% and sheep 7.81%) in Ethiopia. Oryan *et al*. [8] recorded the prevalence of 17.52% in sheep and 55.05% in goats in Fars, Southern Iran and Singh *et al*. [29] reported a prevalence of 4.83% in goats compared to 2.23% in sheep. According to Torgerson *et al*. [10], under conditions of high infestation with *C. tenuicollis*, most sheep develop protective immunity early in life, whereas goats develop protective immunity more slowly. This considerable degree of immunity against *C. tenuicollis* in sheep may explain the reason for the low prevalence of the parasite in sheep.

The prevalence of *C. tenuicollis* in this study was also higher than the reports from other tropical countries in Africa. For instance, in Egypt, a prevalence of 34.5% of *C. tenuicollis* in sheep [30] was reported; in Nigeria, a prevalence of 21.4% in sheep and 34.2% in goats [31] were reported. In Iran, a prevalence of 34.2% in goats, 21.4% in sheep [32] was reported. The prevalence rate differences between studies could be due to the variation in temperature, environmental condition, the degree of pasture contamination because of uncontrolled dog movements and the way of raising and grazing of these animals that may contribute to the transmission cycle between ruminants, dogs and other wild canids. The grazing behavior and management can be considered as the major reasons for this regional difference. Uncontrolled dogs on grazing land as well as in the paddocks, add greatly to the prevalence of this parasite.

With regards to concurrent infections, the individual prevalence of *hydatid* and *C. tenuicollis* cysts was 9.4% and 23.3%, respectively, suggesting that many slaughter ruminants are harboring multiple spp. indicating the need for routine de-worming against internal parasites. Collection of cysts to characterize etiology agent strain and to establish the viability and fertility of hydatid cysts were not determined due to logistic problems.

## Conclusion

First and foremost, the study has shown that tapeworm metacestodes, particularly *C. tenuicollis*, and hydatid cysts are highly prevalent (51.7% and 19.4%, respectively) in Ngorongoro livestock-wildlife interface system, with small ruminants located in Malambo village presenting higher prevalence than in Waso and Endulen. Therefore, with regard to the high prevalence of *Echinococcus* and *Taenia* spp. metacestodes infections found, it is strongly recommended that the current status of hydatidosis infection in humans is properly investigated in the study area and that a joint one health solution on the risks posed by tapeworm infection is adopted.

## Authors’ Contributions

MBM (Livestock Office Ngorongoro) - conceive, design, collect data, analyze and prepared the manuscript. AAK (SUA Morogoro) provided guidance throughout the study period and prepared the manuscript. ESS (Directorate of Veterinary Services) was responsible for design, data analysis and preparation of the manuscript. All authors read and approved the final manuscript.
